# Pathophysiological Significance of WDR62 and JNK Signaling in Human Diseases

**DOI:** 10.3389/fcell.2021.640753

**Published:** 2021-04-16

**Authors:** Yiqiang Zhi, Xiaokun Zhou, Jurui Yu, Ling Yuan, Hongsheng Zhang, Dominic C. H. Ng, Zhiheng Xu, Dan Xu

**Affiliations:** ^1^College of Biological Science and Engineering, Institute of Life Sciences, Fuzhou University, Fuzhou, China; ^2^Center for Medical Genetics, School of Life Sciences, Central South University, Changsha, China; ^3^Fujian Provincial Key Laboratory of Neurodegenerative Disease and Aging Research, Institute of Neuroscience, School of Medicine, Xiamen University, Xiamen, China; ^4^Faculty of Medicine, School of Biomedical Science, University of Queensland, St. Lucia, QLD, Australia; ^5^State Key Laboratory of Molecular Developmental Biology, CAS Center for Excellence in Brain Science and Intelligence Technology, Institute of Genetics and Developmental Biology, Chinese Academy of Sciences, Beijing, China; ^6^Fujian Key Laboratory of Molecular Neurology, Institute of Neuroscience, Fujian Medical University, Fuzhou, China

**Keywords:** WDR62, JNK signaling, neurological disorders, reproductive diseases, tumorigenesis

## Abstract

The c-Jun N-terminal kinase (JNK) is highly evolutionarily conserved and plays important roles in a broad range of physiological and pathological processes. The WD40-repeat protein 62 (WDR62) is a scaffold protein that recruits different components of the JNK signaling pathway to regulate several human diseases including neurological disorders, infertility, and tumorigenesis. Recent studies revealed that WDR62 regulates the process of neural stem cell mitosis and germ cell meiosis through JNK signaling. In this review we summarize the roles of WDR62 and JNK signaling in neuronal and non-neuronal contexts and discuss how JNK-dependent signaling regulates both processes. WDR62 is involved in various human disorders *via* JNK signaling regulation, and may represent a promising therapeutic strategy for the treatment of related diseases.

## Introduction

c-Jun N-terminal kinases (JNKs) form a major signaling cassettes of the mitogen-activated protein kinase (MAPK) pathway. They are highly conserved through evolution (Chang and Karin, 2001). In mammals, the JNK family consists of three genes: *Jnk1*, *Jnk2*, and *Jnk3*, exhibiting more than ten isoforms. The protein sequences of different JNKs share highly similarity; however, they exit different expression patterns (Yamasaki et al., 2012). JNK acts *via* its sequential activation by two upstream regulators, MAPK kinase (MKK) 4 and MKK7. It plays a role in various cellular process including cell proliferation, differentiation, survival and apoptosis (Widmann et al., 1999). Furthermore, it mediates a series of biotic and abiotic stress responses, including infection, inflammation, oxidative stress, and cytoskeleton changes (Zeke et al., 2016).

The WD40 repeat (WDR) domain is among the most abundant protein–protein interaction domains involved in several biological pathways, and that are frequently implicated in disease mechanisms (Schapira et al., 2017). The WD40-repeat protein 62 (WDR62) was first reported as a JNK binding protein that specifically associates with MAPK8/JNK, but not with MAPK1/ERK and MAPK14/p38 (Wasserman et al., 2010). WDR62 contains at least 15 WDR domains, and its deficiency or overexpression are associated with several severe diseases. Mutations in *WDR62* are known to be the second most common cause of autosome recessive primary microcephaly (MCPH) after *ASPM* (Nicholas et al., 2010). Our studies, along with other literatures indicate that WDR62 interacts with different components of the JNK pathway, as well as a E3 ubiquitin ligase, F-box and WD repeat domain-containing protein 7 (Wasserman et al., 2010; Cohen-Katsenelson et al., 2011; Xu et al., 2018). WDR62 is a newly discovered participant in cortical neurogenesis and germ cell-specific division *via* JNK participation (Xu et al., 2014, 2018; Zhou et al., 2018). In this review, we summarize the biological function of WDR62 and JNK signaling, and discuss how JNK-dependent signaling regulates different cell types and related diseases.

## Expression Profile of WDR62 and JNKs

### Cellular Localization of WDR62 and JNKs

Several studies claimed that WDR62 exhibits cell type and cell cycle-dependent expression. In interphase cells, WDR62 exhibits weak, and diffuse cytoplasmic expression. During mitosis, WDR62 accumulates strongly at the spindle poles (Nicholas et al., 2010). The association of WDR62 with spindle microtubules in the vicinity of separated centrosomes increases rapidly following mitotic entry (Kodani et al., 2015). One study on the endogenous expression pattern of WDR62 in human and mouse embryonic brain revealed cytoplasmic expression of mitotic apical and basal neural precursor cells and nuclear localization in new born neurons in the cortical plate (Nicholas et al., 2010). In contrast, Bilguvar et al. (2010) showed that WDR62 predominantly accumulates in the nucleus and co-localizes with an apical progenitor marker, SOX2, in cortical slices of the mice embryonic brain. Moreover, Yu et al. (2010) demonstrated perinuclear localization and co-localization with the Golgi apparatus of WDR62 in interphase HeLa cells.

The intracellular distribution of JNK family members is diverse and subject to regulation by complex mechanisms. The majority of cellular JNK is soluble and present in both cytoplasm and the nucleus. Interestingly, insoluble fractions of JNK are localized in nuclear and cytoplasmic speckles and within centrosomes (MacCorkle-Chosnek et al., 2001). The co-expression of JNKs (JNK1/JNK2/JNK3) with WDR62 protein results in co-localization of JNKs and WDR62 in the cytoplasmic granules from predominate nuclear localization of JNKs without WDR62 (Wasserman et al., 2010). β-arrestin 2, also known as arrestin 3, is reportedly a binding partner of JNK3. The subcellular distribution of JNK3 was regulated by nucleocytoplasmic shuttling of β-arrestin 2 (Scott et al., 2002). JNK is activated by external stresses, including changes in osmolarity, heat shock, and UV irradiation. Under hyperosmotic stress, the dynamics of both cytoplasmic and nuclear JNK1 slows, and the entry of JNK1 into the nucleus is restricted (Misheva et al., 2014). In response to heat stress, JNK1 promotes the translocation of DAF-16, a forkhead transcription factor, into the nucleus (Oh et al., 2005). Under vacuum stress, the survival of HEK-293 cells is attributed to the production of endogenous cytokines, based on the NF-kB signaling pathway, and the continuous activation of JNK in mitochondria (Jack et al., 2006). In HCT116 cells, high glucose, insulin, and palmitic acid triggered the centrosome amplification and enhance the expressions of JNK1 and signal transducer and activator of transcription 3 (Stat3) (Lu et al., 2019). These results indicate the important role of JNKs especially JNK1 in the cellular stress response.

Furthermore, activated JNK have been detected in cultured cells not subject to stress and isolated mouse tissues, such as the brain, indicating the importance of JNK signaling in physiological processes. During mitosis, JNK1, but not JNK2, is recruited to the spindle pole by WDR62 in HEK-293 cells (Lim et al., 2015). Studies in mouse oocytes revealed the association of JNK2 with spindle poles during meiosis (Huang et al., 2011). In *Drosophila* intestinal stem cells, p-JNK and WDR62 exhibit mutual dependence in the recruitment to the spindle (Hu and Jasper, 2019). In retinal progenitor cells, JNK is phosphorylated during the early stages of mitosis (Ribas et al., 2012). In HEK-293 cell, JNK phosphorylation negatively regulates WDR62 binding to microtubules (Lim et al., 2015). Other studies showed JNK-mediated phosphorylation of the WDR62 in C-terminal is not necessary for spindle localization, whereas it is required to maintain the function of WDR62 in the mid-spindle structure (Bogoyevitch et al., 2012). These results suggest that WDR62 and JNKs have diverse roles in different cell types and cell conditions.

### WDR62 and JNK1 Transcriptional Expression in Developing Human Brains

**FIGURE 1:**
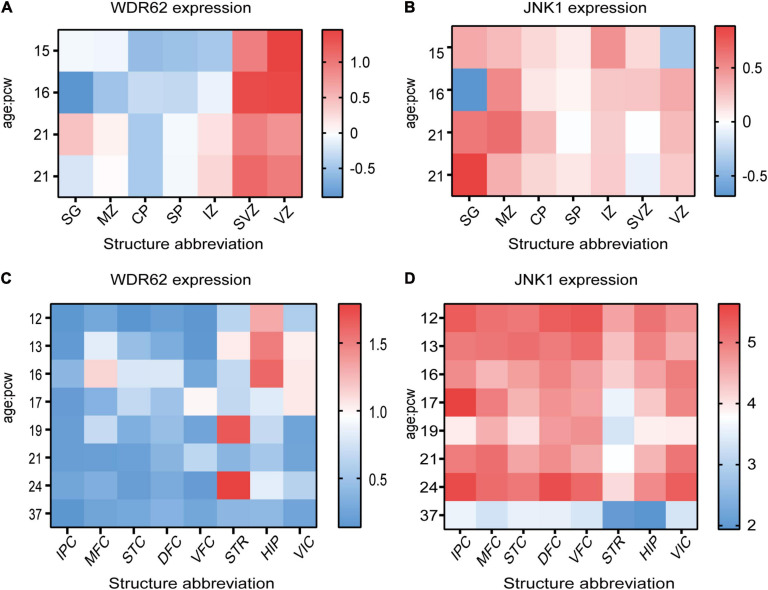
WDR62 and JNK1 expression during human brain development. **(A,B)** Heatmap showing the expression levels of WDR62/JNK1 in different cortical layers of cortex at post-conception weeks (PCW) 15, 16, and 21. **(C,D)** The transcriptional expression of WDR62/JNK1 in different regions of human brains at PCW 12–37. Two different samples (male and female) are depicted in the expression of WDR62 and JNK1 in different layers of cortex. Red depicts high expression, and blue depicts low expression. CP, cortical plate; DFC, dorsolateral prefrontal cortex; HIP, hippocampus; IPC, inferior parietal cortex; IZ, intermediate zone; MFC, medial prefrontal cortex; MZ, marginal zone; SG, subpial granular zone; SP, subplate zone; STC, caudal superior temporal cortex; STR, striatum; SVZ, subventricular zone; VFC, ventrolateral prefrontal cortex; VIC, primary visual cortex; VZ, ventricular zone.

The centrosome association of WDR62 and JNK1 indicates JNK1 is more dominant related with WDR62 compared with JNK2 or JNK3 in localization. We investigated the expression pattern of WDR62 and JNK1 during the central nervous system development based on the data from the BrainSpan Atlas. During the fetal period (post-conceptual weeks (PCW) 15–21), WDR62 exhibited high expression levels in the ventricular zone (VZ) and subventricular zone (SVZ), compared to other cortical layers (Figure 1A). This is consistent with the important roles of WDR62 in neural progenitor cell (NPC) proliferation and differentiation. However, JNK1 had widespread expression from VZ to the subpial granular zone in the cortex (Figure 1B). This implies a greater diversity of functions for JNK1 which is not restricted to NPCs. WDR62 expresses at a quiet low level during PCW 12–37 across the different brain regions. It shows relatively higher expression at PCW 12–16 in the hippocampus and PCW 19 or 24 in the striatum, compared to other regions (Figure 1C). The JNK1 expression in different regions of brain is relatively high. A sharp decrease at the full term of PCW37 indicates the importance of JNK1 in the embryonic stage (Figure 1D).

## Roles of WDR62 and JNK Signaling During Mitosis and Meiosis

### WDR62 Is Associated With Spindle Pole During Mitosis

The specific cellular localization of WDR62 and JNKs dictate their roles in the regulation of mitosis and meiosis. Centrosomes are embedded within the poles of the mitotic spindle and act as microtubule organizing centers (MTOCs) (Meraldi, 2016). WDR62 is required for spindle pole formation and its depletion results in the reduced spindle-centrosome attachment (Bogoyevitch et al., 2012). In the late stage of mitosis, WDR62 interacts with Aurora A (AURKA) to regulate spindle formation, mitotic progression and brainsize (Chen et al., 2014). Furthermore, the expression of WDR62 is closely related to the composition of the chromosome passenger complex, which is a key regulator of mitosis (Sgourdou et al., 2017). WDR62 has been shown to be a substrate for the mitotic kinase, Polo-like kinase 1, which phosphorylates human WDR62 on Ser 897. This phosphorylation at the spindle poles promotes astral microtubule assembly to stabilize spindle orientation (Miyamoto et al., 2017). In *Drosophil*a, the loss of an active MTOC in *Wdr62* mutants compromises centrosome positioning, spindle orientation, biased centrosome segregation, and the cell cycle which leads to a reduced brain size (Ramdas Nair et al., 2016). In mice, lower expression of WDR62 results in a multipolar spindle, unstable spindle structure, mitotic arrest and cell death (Chen et al., 2014; Xu et al., 2014). The spindle pole localization of WDR62 and mitotic progression are obviously defective in patient-derived fibroblasts, which become transiently arrested at the prometaphase (Sgourdou et al., 2017). These results reinforce importance of WDR62 in mitosis regulation.

### JNK Control the Onset of Mitosis

Similarly to WDR62, JNK is regulated in a cell cycle dependent manner (Gutierrez et al., 2010a). JNK phosphorylation increases at G2/M phase, and JNK-specific inhibitor SP600125 blocks mitotic entry (Lee and Song, 2008). JNK activity regulation results in impaired entry into mitosis and abnormal spindle and chromosomal dynamics. In human melanoma cells, JNK inhibition induces either predominant G2/M arrest or apoptosis depending on the cell line (Alexaki et al., 2008). In human primary fibroblast IMR90 cells, JNK promote mitotic progression through histone H3 phosphorylation (Lee and Song, 2008). During G2 and early mitosis, JNK phosphorylates Cdh1 directly, independently of cyclin-dependent kinases (CDKs) activation (Gutierrez et al., 2010a). However, JNK directly phosphorylates Cdc25C during the G2 phase, and negatively regulates CDK1 activation (Gutierrez et al., 2010b). JNK is also found to promote mitosis through Aurora B kinase (Oktay et al., 2008). These results indicate that JNK control the onset of mitosis by mediate phosphorylation of different substrates.

### WDR62 and JNK Signaling Are Involved in Meiosis

Meiosis is the formation process of the egg and sperm cell. In female meiosis, WDR62 is responsible for asymmetric meiotic division during mouse oocyte maturation (Wang et al., 2020). The inhibition of WDR62 affects the initiation of meiosis, such that most germ cells in ovaries are lost during embryonic stage (Qin et al., 2019). Interestingly, the germ cell loss can be partially rescued by the overexpression of JNK1 (Zhou et al., 2018). The JNK signal (JNK1/JNK2) is also likely to be involved in the regulation of bovine oocyte maturation, which might be independent of the M-phase promoting factor activity and meiotic resumption (Vigneron et al., 2004). Moreover, JNK2 likewise plays an important role in spindle assembly and first polar body extrusion during oocyte meiotic maturation (Huang et al., 2011). Both at metaphase I and metaphase II stages, JNK2 was associated with the MTOCs within the spindle poles. In spermatogenesis, WDR62 is required for germ cell meiosis initiation. However, downregulation of the *Vdac2* gene inhibits spermatogenesis *via* the JNK/P53 cascade (Fang et al., 2020). Another study found that JNK signaling regulates germ cell apoptosis through activation of p53 and suppression of survival during testicular ischemia reperfusion injury (Fadel et al., 2020).

## WDR62 in Neurological Diseases

WDR62 was identified as the second most causative gene of MCPH that exhibits additional neurological deficits in human patients (Table 1). *Wdr62* mutations in humans include severe cortical malformation, cerebellum, corpus callosum and ventricular dysplasia, and as well as neurological complications such as epilepsy (Bilguvar et al., 2010; Poulton et al., 2014; Zombor et al., 2019). A recent large-scale whole-genome sequencing study found numerous microcephaly-related gene mutations in patients with autism spectrum disorder, including *WDR62*, *ASPM* and *ZNF335* (Li et al., 2017). The missense mutation of *WDR62* (c.1949G > A, p.R650H) which is located in the WD repeat b domain of the WDR62 protein is a possible candidate for ASD (Wu et al., 2018).

**TABLE 1:** WDR62 and JNK signaling in neurological disorders.

**Gene**	**Mutations**	**Types of disease**	**Phenotypes**	**References**
WDR62 (NM_001083961.2)	c.28G > T (p.A10S) c.189G > T (p.E63D) c332G > C (pR111T) c.363delT (p.D112Mfs*5) c.390G > A (p.E130 E) c.535_536insA (p.M179fs*21) c.671G > C (p.W224S) c.668T > C (p.F223S) c.731C > T (p.S244L) c.797C > T (p.A266V) c.900C > A (p.C300*) c.1043 + 1G > A (p.S348Rfs*63) c.1102G > A (p.D368N) c.1143delA (p.H381PfsX48) c.1194G > A (p. W398*) c.1313G > A (p.R438H) c.1408C > T (p.Q470*) c.1531G > A (p.D511N) c.1576G > T (p.E526*) c.1576G > A (p.E526K) c.1605_1606insT (p.E536*) c.1684C > G (p.H562D) c.1942C > T (p.Q648*) c.1949G > A (p.R650H) c.2083delA (p.S696Afs*4) c.2115C > G (p.G705G) c.2195C > T (p.T732I) c.2413G > T (p.E805X) c.2472_2473delAG (p.Q918Gfs*18) c.2864-2867delACAG (p.D955Afs*112) c.2867 + 4_c2867 + 7delGGTG (p.S956Cfs*38) c.3232G > A (p.A1078T) c.3361delG (p. A1121Qfs*6) c.3503G > A (p.W1168*) c.3839_3855del (p.G1280Afs*21) c.3878C > A (p.A1293D) c.3936dupC (p.V1314Rfs*18) c.4241dupT (p.S1415Efs*40)	MCPH	Reduced brain size, pachygyria corpus, callosum abnormalities, schizencephaly, hippocampal and cerebellar hypoplasia	Miyamoto et al., 2017; Naseer et al., 2019; Rasool et al., 2020; Shohayeb et al., 2018; Zombor et al., 2019
WDR62 (NM_001083961.2)	c.3406C > T (p.R1136*)	ARID	Impairment in cognitive ability and adaptive behavior	McSherry et al., 2018
	c.1949G > A (p.R650H) c.4354-4356AGA > TGG (p.R1452W)	ASD	Social disorders, speech disorders and awkward motor behavior	Wu et al., 2018
	c.1821dupT (p.R608Sfs*26) c.2584G > A (p.G862S); c.2859_2862delACAG (p. N955Afs114*)	Epilepsy	Lissencephaly	Kolbjer et al., 2021; McDonell et al., 2014

**JNK activity**	**Methods of detection**	**Tissue analyzed**	**Tissue analyzed**	**References**

Up	Western blot immunostaining	Dopaminergic neurons from PD patients and MPTP mouse model of PD.	PD	Hunot et al., 2004
Up	Western blot immunostaining	Ventral midbrain of MPTP mouse model	PD	Huang et al., 2016
Up (JNK3)	Western blot	Cellular and mouse models of Huntington’s disease	HD	Morfini et al., 2009
Up	Western blot	Striatal cultures co-expressing polyglutamine-expanded huntingtin	HD	Taylor et al., 2013
Up	Western blot	Postmortem tissue of patients	Ischemic stroke	Mitsios et al., 2007
Up (JNK/JNK3)	Western blot immunostaining	Brain and cerebrospinal fluid of patients	AD	Gourmaud et al., 2015
Up	Western blot	LPS induce rat brains	Depression like behavior	Zhang et al., 2020
Up	Western blot	Nucleus and the post-synaptic protein-enriched fraction of Ube3am^–/p+^ mice	AS	Musi et al., 2020

AD, Alzheimer’s disease; AS, Angelman syndrome; ARID, autosomal recessive intellectual disability; ASD, Autism spectrum disorder; HD, Huntington Disease; LPS, Lipopolysaccharide; MCPH, Autosomal recessive primary microcephaly; MPTP,1-methyl-4-phenyl-1,2,4,6-tetrahydropyridine; PD, Parkinson’s Disease. *means stop codon.

### WDR62 Control Brain Size by Regulating Neural Progenitor Cells

**FIGURE 2:**
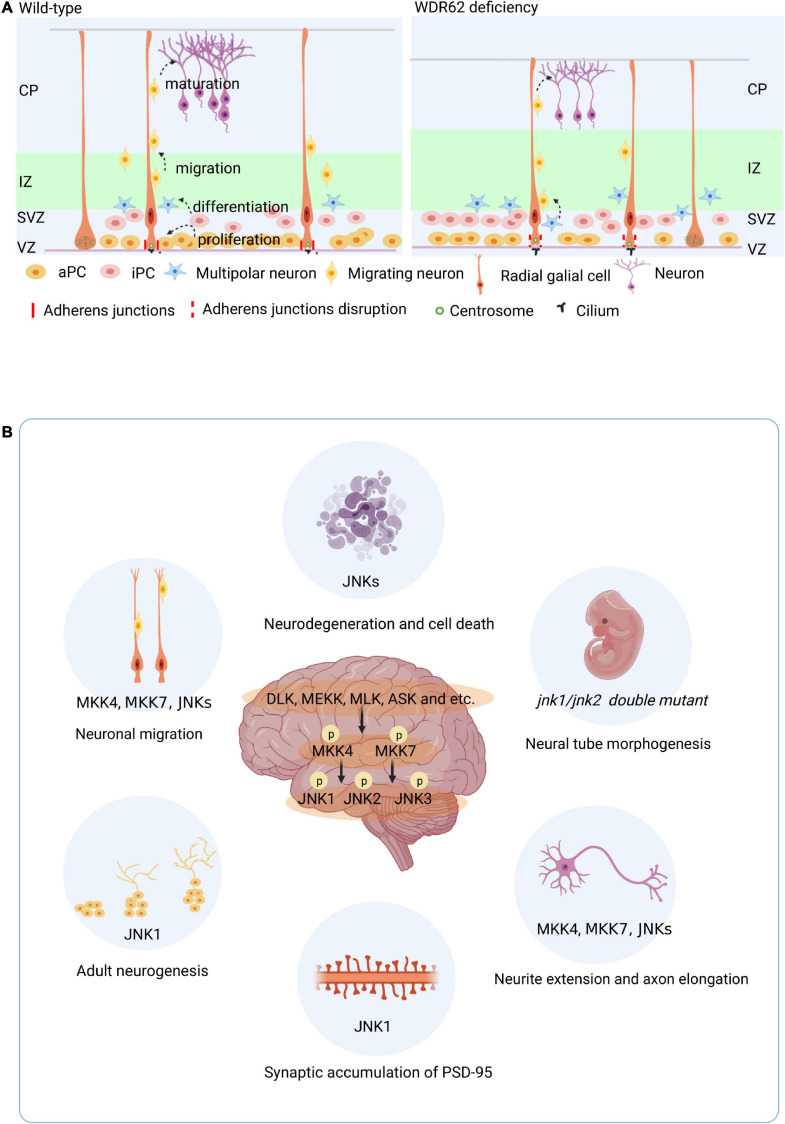
Roles of WDR62 and JNK signaling in brain. **(A)** WDR62 control of embryo brain cortical neurogenesis. Development of the mammalian cortex requires precise timing of proliferation/self-renewal of neural progenitor cells (NPCs) differentiation, neuronal migration, and maturation. WDR62 depletion leads to premature differentiation of neural progenitor cells with long cilia and disrupted adhesion junction proteins in the VZ. **(B)** Diverse role of JNK signaling in the brain. aPC, apical precursor cell; CP, cortical plate; iPC, intermediate precursor cell; IZ, intermediate zone; SVZ, subventricular zone; VZ, ventricular zone.

Development of the mammalian cortex requires precise timing of proliferation/self-renewal of neural progenitor cells (NPCs), differentiation, neuronal migration, and maturation (Figure 2A). The proliferation and differentiation of NPCs mainly take place in the VZ and SVZ, respectively (Noctor et al., 2004). NPCs increase the progenitor cell pool by symmetrical division, and generate progenitor cells (maintaining the progenitor cell pool) and a nerve precursor through asymmetrical division (Paridaen and Huttner, 2014). Eventually, NPCs differentiate to neuron which migrate and differentiated to form the cortex (Figure 2A). The disturbance of the symmetric cell divisions can lead to a reduction of the neural progenitor pool, a subsequent decrease in proliferation, and a decline of neuron production levels. The end result is a smaller than usual brain size or microcephaly.

WDR62 also known as MCPH2 reinforces the important of its role in the developing mammalian brain. The enrichment of WDR62 in the VZ and SVZ of the developing human brain indicates its role in embryo neurogenesis (Figure 1A). WDR62 depletion in NPCs inhibits their self-renewal and induces premature differentiation, which may be related to retarded cilium disassembly, long cilium and delayed cell cycle progression in microcephaly (Xu et al., 2014; Zhang et al., 2019). Moreover, WDR62 is required to ensure normal adhesion of the junction protein in apical complex construction (Jayaraman et al., 2016; Figure 2A). A recent study showed that WDR62 is required for neurogenesis and the growth of the hippocampus during embryonic development (Shohayeb et al., 2020a). Endogenous expression of WDR62 was detected during the induction and development of human pluripotent stem cells into NPCs and glial cells. The content of WDR62 increased at the early induction stage and decreased significantly toward the end (Alshawaf et al., 2017), which indicates the level of WDR62 expression influences the differentiation of neurons.

### Glial Specific Functions of WDR62

Glial cells include astrocytes, oligodendrocytes, and microglia, all of which are essential parts of the nervous system. In *Drosophila* melanogaster, decreased WDR62 expression in neural stem cells lowered the numbers of neural stem cells, but did not affect the brain size. In contrast, glial specific WDR62 depletion significantly reduced brain volume, not only by decreasing the glial population, but also non-autonomously causing neuroblast loss (Lim et al., 2017). Accordingly, glia specific WDR62 overexpression in of *Drosophila* larval brains promotes brain overgrowth, without overt disruption of the tissue organization (Shohayeb et al., 2020b). Both studies demonstrate WDR62 level control of *Drosophila* brain growth through mitotic kinase AURKA signaling (Lim et al., 2017; Shohayeb et al., 2020b).

## Diverse Roles of JNK Signaling in Different Neurological Disorders

### Neuronal Function of JNK Signaling

Brain phenotypes of different knockout mice show that JNK signaling regulates diverse processes in the brain (Figure 2B). Both JNK1 and JNK2 play roles in neural precursors and are required for proper neural tube morphogenesis (Sabapathya et al., 1999). During embryonic stem cell neurogenesis, the *Jnk1* knock out (KO) increased the expression of WNT-4 and WNT-6, which are inhibitors of neurogenesis (Amura et al., 2005). Moreover, metabotropic glutamate receptors promote the proliferation of human embryonic cortical NPCs by increasing p-JNK2 levels (Guo et al., 2014). JNK signaling not only plays important roles in embryonic development, brain morphogenesis, and neuronal pathfinding, but also in the adult brain, which retains a high level of JNK expression. In contrast to embryo stage, *Jnk1* KO mice showed increased neurogenesis in the adult hippocampus, characterized by enhanced cell proliferation and survival, and increased maturation in the ventral region (Mohammad et al., 2018). Both J*nk2 ^–/–^* or *Jnk3 ^–/–^* mice showed robustness in the face of neuronal stress induced apoptosis, which differs from *Jnk1 ^–/–^* (Castro-Torres et al., 2019). The activators of JNK isoforms MKK4 and MKK7 are required for neuronal migration and axon maintenance or elongation in the developing brain (Wang et al., 2007; Yamasaki et al., 2011). In addition, neuron-specific *Mkk7* knockout mice displayed age-dependent motor dysfunction (Yamasaki et al., 2017). JNK phosphorylates doublecortin at S332 to modulate neurite extension and neuronal migration *in vivo* (Jin et al., 2010). The Rac1-JNK1 signaling pathway mediates ser-295 phosphorylation and regulates synaptic accumulation of PSD-95 (Kim et al., 2007). In general, MKK4, MKK7, and the JNKs contribute to the regulation brain development through both overlapping and non-redundant mechanisms. JNK1 is dominating isoform in the regulation of neurogenesis both in developing and adult brain.

### JNK Signaling in Glial Cell

The role/impact of JNK signaling in astrocytes has been well documented. Astrocytes have significant structural, metabolic and trophic roles in modulating and coordinating neuronal structure and function, both in normal brain function and in disease states (Araque and Navarrete, 2010). Endothelin-1 not only inhibits neural progenitor cell migration, but also regulates astrocyte proliferation and reactive gliosis *via* a JNK/c-Jun signaling pathway (Gadea et al., 2008). Cocaine-induced JNK activation leads to the up-regulation of cyclin A2 and the enhanced proliferation of human astrocytes (Lee et al., 2016). Activation of the MMP2-JNK1/2 signaling pathway in astrocytes has been shown to contribute to the pathogenesis of pain hypersensitivity in the chronic post-ischemia pain model (Tian et al., 2017). The protein zero (P0) glycoprotein is a vital component of compact peripheral nerve myelin produced by the glial cells of the mammalian peripheral nervous system. One study showed that JNK1 activation mediates P0 mRNA instability and transcriptional repression in Schwann cells (David et al., 2006). Moreover, Aβ exposure or traumatic injury elicits JNK activation in astrocytes (Augustine et al., 2014; Li et al., 2018). These results suggest that JNK activation may contribute to neurological diseases mediated by astrocytes.

### JNK Regulation in Psychiatric Diseases

Normal JNK activity is important for neuronal development. Excess activation of JNK will induce neurodegeneration and pathological cell death associated with neurodegenerative diseases. JNK and p38 are critical for the activation of secreted forms microglia by β-amyloid precursor protein, a process that compromises neuronal function and survival (Bodles and Barger, 2005). Furthermore, JNK activity down regulation likewise gives rise to cortical defects. The inhibition of JNK accelerates radial migration and leads to ill-defined cellular organization (Westerlund et al., 2011).

Excitotoxicity insults can induce JNK activation, which leads to neuronal death and contributes to numerous of neurological diseases, such as cerebral ischemia and neurodegenerative disorders (Spigolon et al., 2010). JNK is activated in neurons in Alzheimer’s disease (AD), Parkinson’s disease (PD), stroke, polyglutamine disease, etc. (Table 1). Activation of the JNK signaling pathway is involved in the deposition of AD and its induced neurotoxicity. The JNK inhibition by an inhibitor or electroacupuncture exhibited neuroprotective effects on Aβ-induced memory impairment and apoptosis (Ramin et al., 2011; Tang et al., 2020). JNK inhibition is likewise a potential treatment for PD (Hunot et al., 2004), as JNK is activated in PD and various toxicant models of the disease and implicated in associated dopamine-induced neuron apoptosis (Peng and Andersen, 2003).

JNK3 is mainly expressed in the brain tissue, and it is the isoform most responsive to stress-stimuli. JNK3 activation has a key role in triggering apoptosis (Lin et al., 2017) and neuronal death in several neurodegenerative disorders, such as AD and PD (Antoniou et al., 2011). Inhibiting the ASK1-JNK3 pathway by disrupting the interaction between β-arrestin-2 and JNK3 has been revealed as an effective strategy for preventing dopaminergic neuron loss in PD (Pan et al., 2015). In optic neuropathies, JNK3 expression and activation are upregulated in retinal ganglion cells after optic nerve axotomy, leading to retinal ganglion cell degeneration (Liu C. et al., 2017). An *in vivo* study showed that complexes of MKK4 or MKK7 with JNK3 increase in rat brain mitochondria, while MKK4:JNK1 complexes decrease following transient middle cerebral artery occlusion (Zhao and Herdegen, 2009).

JNK regulation is also vital in emotional disorders, such as depression, and schizophrenia. Studies identified that the inhibition of JNK by JNK1 knockout or inhibitors increases the number of adults born granule cells in the hippocampal neurogenic niche, while alleviating anxiety and reducing depressive behaviors (Mohammad et al., 2018). In accordance, JNK inhibition ameliorates neuroinflammation-induced depressive-like behavior (Zhang et al., 2020). Haploinsufficiency of Map2k7, a JNK activator, induces brain imaging endophenotypes and behavioral phenotypes relevant to schizophrenia (Openshaw et al., 2020).

## WDR62 and JNK Signaling in Reproductive System Diseases

Because WDR62 and JNK signaling both function in mitosis and meiosis, it is not surprising that the deregulation of WDR62 or JNK activity leads to defects in the development of the reproductive system (Table 2). However, the precise mechanisms by which WDR62 and JNK signaling regulate meiosis have not yet been completely elucidated.

**TABLE 2:** WDR62 and JNK signaling in reproductive systems.

**Gene (mutation/up/down)**	**Mutation/mouse model**	**Types of disease**	**Phenotypes**	**References**
WDR62 (mutation)	c.1796G > A (p.C599Y) c.3203_3206 del (p.T1068fs)	POI	At least four months of hypomenorrhea or amenorrhea, increased levels of FSH and decreased levels of estradiol in women younger than 40 years old	Malheiros and Bilharinho, 2019
WDR62 (deficiency)	WDR62 knock out mice	Infertility	Male germ cells are lost and metaphase I arrest of spermatocytes	Qin et al., 2019; Zhou et al., 2018
WDR62 (down)	*Wdr62* siRNA microinjection in mice	N/A	First polar body extrusion defect	Wang et al., 2020
JNK (down)	JNK inhibition by shRNA or SP600125 treatment	N/A	Abnormal germline cyst breakdown and failed primordial follicle formation	Niu et al., 2016
JNK (down)	JNK inhibitors, SP600125 and AS601245	N/A	granulosa cells cell cycle disruption and follicle growth regression	Oktem et al., 2011
JNK (down)	JNK2 antibody microinjection or SP600125 treatment	N/A	Abnormal spindle formation and decreased the rate of first polar body extrusion	Huang et al., 2011

*POI, Premature ovarian failure; N/A, not applicable.*

### WDR62 and JNK Signaling in Spermatogenesis

**FIGURE 3:**
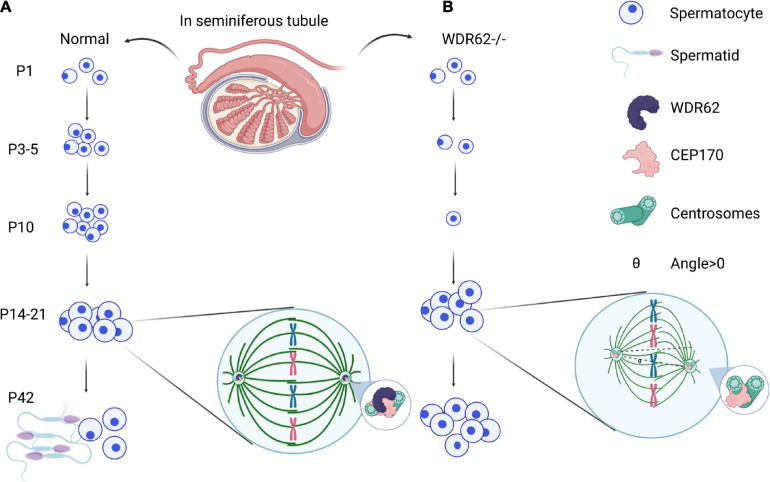
WDR62 in spermatogenesis. **(A)** Male meiosis begins with a cell called a primary spermatocyte. Spermatids are the products of meiosis from the tetraploid primary spermatocytes *via* meiotic cell division. **(B)** WDR62 deficiency resulted in a loss of germ cells at P3, P5 and P10, which was gradually recovered from at P14-P21. During meiosis, the spindle of spermatocytes from *Wdr62* KO mice is disrupted, and the position of the spindle poles is asymmetric. The intensity of α-tubulin is markedly diminished in WDR62 deficient spermatocytes, and the angle of intersection between the polar axis of the main axis and the plane direction (θ) is markedly increased, leading to meiotic arrest. P, postnatal day.

Spermatogenesis in the male reproductive system is a highly specialized process of cellular differentiation to produce spermatozoa (Barroca et al., 2008). Spermatogonial stem cells differentiate into spermatocytes *via* mitotic cell division. Spermatids are the products of meiosis from the tetraploid primary spermatocytes *via* meiotic cell division (Figure 3A). The spindle assembly checkpoint is a key regulator of chromosome segregation in both mitosis and meiosis (Gorbsky, 2015). WDR62 is required for germ cell meiosis initiation in mice, and the majority of male germ cells are lost due to the meiotic defect of first wave spermatogenesis in *Wdr62* mutants (Figure 3B). Furthermore, studies show that WDR62 interacts with centrosome-associated protein CEP170, and the deletion of WDR62 causes downregulation of the CEP170 protein, which in turn leads to the aberrant spindle assembly. The metaphase I arrest of WDR62-deficient spermatocytes is caused by asymmetric distribution of the centrosome and aberrant spindle assembly which leads to defects in spermatogenesis (Qin et al., 2019; Figure 3B).

JNK signaling likewise involved in spermatogenesis. In the *Drosophila* testis, JNK activity is autonomously required for spermatogonial dedifferentiated into germline stem cells (SSC) under chronically stressful conditions (Herrera and Bach, 2018). Further, JNK hyperactivation-mediated glycolysis contributes to SSC aging in mice (Kanatsu-Shinohara et al., 2019). Moreover, germ cell apoptosis is linked to the JNK pathway during testicular ischemia reperfusion injury (Al-Kandari et al., 2019).

### WDR62 and JNK Signaling in Female Meiosis

**FIGURE 4:**
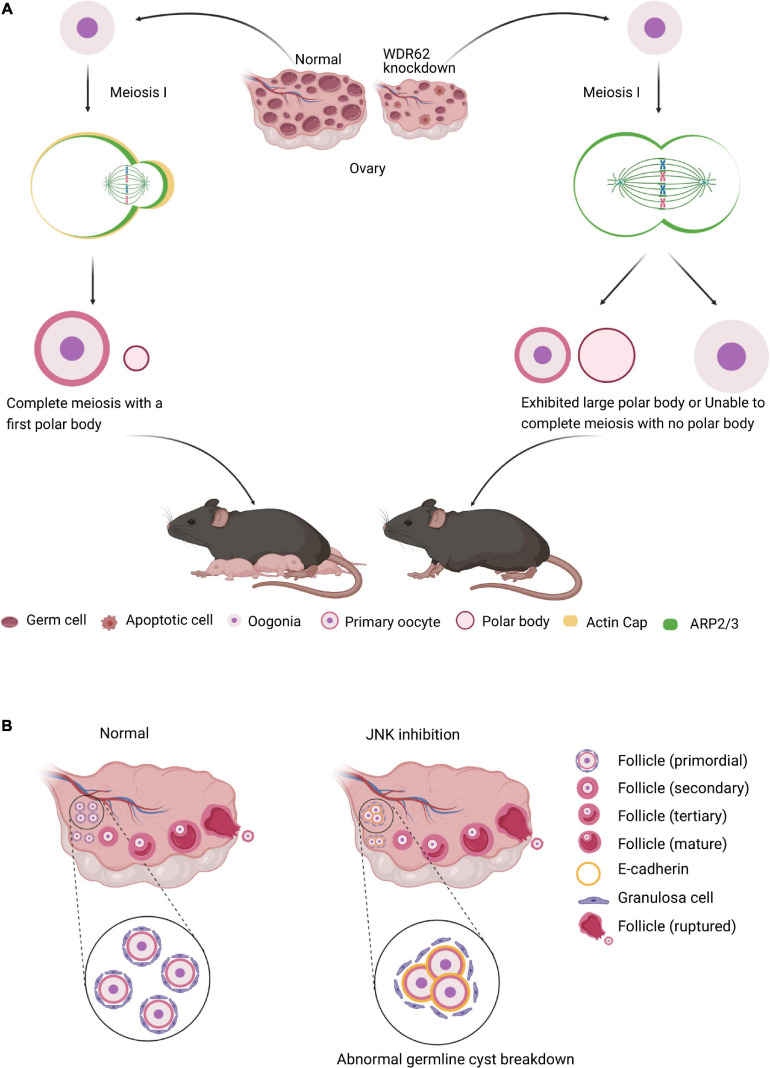
WDR62 and JNK signaling in female meiosis. **(A)** In female meiosis, oocyte meiotic maturation is a form of asymmetric cell division, producing the first polar body and a large oocyte. The knockdown of WDR62 is associated with the apoptosis of germ cells, shrinking of the ovary and causing infertility, possibly by affecting spindle assembly and disrupting extrusion and asymmetric division of the first polar body. This process may require the modulation of ARP2/3 activation as well as the actin cap. **(B)** The JNK signaling is critical for germline cyst breakdown and primordial follicle formation. Disruption of the JNK signaling dramatically inhibits cyst rupture and primordial follicle formation in cultured mouse ovaries. In addition, E-cadherin is robustly expressed in germline cysts and its decline is required for oocyte release from cysts. However, inhibition of JNK signaling results in abnormally enhanced localization of E-cadherin adhesion protein at oocyte-ovarian contact sites and delays cyst rupture.

In female meiosis, oocyte meiotic maturation is a form of asymmetric cell division, producing the first polar body and a large oocyte, in which the asymmetry of the oocyte meiotic division depends on spindle migration and positioning, and cortical polarization (Yi et al., 2013; Figure 4A). WDR62 regulate the distribution of cortical actin and Arp2/3 complex during mouse oocyte maturation. The knock down of WDR62 disrupts cytoskeletal of spindle organization and migration which affects the first polar body extrusion and asymmetric division (Figure 4A; Wang et al., 2020). This may identify the *WDR62* mutation in human as a potential cause of premature ovarian failure (POI).

Several studies indicate that *Wdr62* KO mice germ cell is largely disrupted, which is dependent on JNK activity. In mammalian oocyte meiosis, JNK plays very similar role to WDR62. JNK2 participates in spindle assembly and first polar body extrusion (Huang et al., 2011). Inefficient assembly of the primordial follicle and the primordial follicle transition to the primary follicle are the two main processes contribute to ovarian pathologies (Skinner, 2005). The JNK activity is activated in mitotic granulosa cells of follicles and required for preanatal follicle growth (Oktem et al., 2011). JNK is specifically localized in oocytes and regulates E-cadherin expression at germline cysts (Figure 4B). The downregulation of JNK signaling with inhibitor SP600125 or shRNA results in aberrant E-cadherin junctions, abnormal germline cyst breakdown and unsuccessful primordial follicle formation (Figure 4B; Niu et al., 2016). These results demonstrate that both WDR62 and JNK signal play important roles in oocyte meiotic division.

## WDR62 and JNK Signaling Are Implicated in Tumorigenesis

Neurogenesis and tumorigenesis share common molecular principles regulating key processes associated with cell division and apoptosis. Several studies have linked WDR62 and JNK signaling with tumorigenesis (Wu et al., 2019; Zhou et al., 2020).

### WDR62 as a Potential Biomarker for Cancers

Most *MCPH* genes including *WDR62* are associated with centriole over-duplication, multipolar spindles, anaphase-lagging chromosomes, and micronuclei. This implies the crucial role of *MCPH* genes in tumorigenesis. *WDR62* mRNA level is significantly overexpressed in lung adenocarcinoma and a multivariate analysis revealed that *WDR62* overexpression is an independent predictor of a poor survival outcome among lung adenocarcinoma patients (Shinmura et al., 2017). WDR62 is overexpressed in most of the epithelial ovarian cancer cell lines and tumors, especially in high-grade carcinoma of the ovary (Zhang et al., 2013). Other study implies WDR62 overexpression is an important molecular change, specifically related to ovarian cancer with centrosome amplification. It may play a role in both tumor initiation and progression (Zhang et al., 2013). Similarly, WDR62 expression is significantly increased in gastric cancer tissues and cell lines, and it is associated with poor differentiation and prognosis as well as multidrug resistance in gastric cancer. Accordingly, suppressing WDR62 significantly inhibits cell proliferation and induces the G2/M phase arrest of gastric cancer cells (Zeng et al., 2013). Thus, WDR62 can be considered as a potential biomarker for the detection and differentiation grade of various cancers.

### Pro and Anti-oncogenic Roles of JNK Signaling in Cancers

**FIGURE 5:**
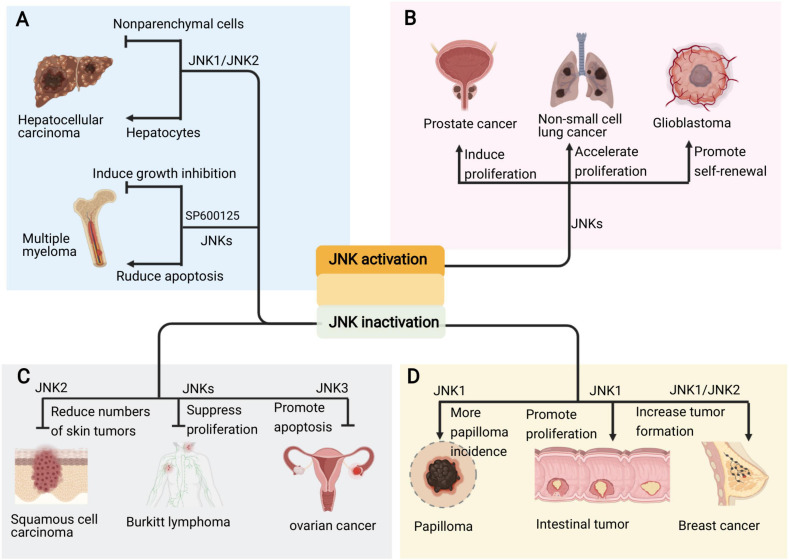
Pro and anti-oncogenic roles of JNK signaling in cancers. **(A)** The JNK signaling plays due role in the occurrence of different kind of cancers. **(B)** Sustained activation of JNKs promotes the development and progression of cancers such as prostate cancer, non-small cell lung cancer and glioblastoma. **(C)** Inhibition of different JNKs also suppresses skin cancer, lymphoma and ovarian cancer. **(D)** However, deficiency or inhibition of JNK, especially JNK1, also cause intestinal tumor, papilloma and breast cancer.

The specific role of JNKs in tumorigenesis is complex. The consequences of JNK signaling can have opposing outcomes depending on tumor context (Figure 5). For example, JNK signaling is able to both eliminate pre-tumorigenic cells *via* apoptosis, whereas it also collaborate with various genetic insults to stimulate tumorigenesis (La Marca and Richardson, 2020). The inhibition of JNK activity inhibition by SP600125 induces growth inhibition *via* cell cycle arrest and activates NF-κB in the multiple myeloma (MM) cell line (Hideshima et al., 2003). However, other study indicate SP600125 abrogated PKC412-induced apoptosis in the MM cell line (Sharkey et al., 2007; Figure 5A). Compound deficiency of JNK1 and JNK2 indicate that JNK plays a duel role (i.e., tumor promoting and suppressing) in the development of hepatocellular carcinoma (HCC), which is cell type dependent (Das et al., 2011; Wu et al., 2019). The deletion of JNK1 in hepatocytes and JNK2 in the entire organism fueled the development of HCC, which indicates that JNK activity works to inhibit tumor development. However, in non-parenchymal cells of the liver, JNK activity is reported to promote HCC development by expressing protumorigenic cytokines (Figure 5A). Moreover, a study in human pancreatic cancer cell lines showed that JNK1 and JNK2 exert different functions in human pancreatic cancer (Tian et al., 2021).

Emerging evidence implies that the persistent activation of JNKs is involved in cancer development and progression supporting the pro-oncogenic role of JNK in prostate cancer, non-small cell lung cancer, and glioblastoma, among others (Figure 5B; Luo et al., 2019; Portela et al., 2019; Xu and Hu, 2020). Accordingly, JNK inactivation inhibits tumorigenesis in skin cancer, lymphoma and ovarian cancer (Figure 5C; Yang et al., 2015; Ding et al., 2020; Hammouda et al., 2020). Therefore, the JNK signaling represents attractive targets for therapeutic intervention with small molecule kinase inhibitors.

Studies on JNK deletion in various mouse cancer models show that JNK acts as a tumor suppressor (Cellurale et al., 2010; Schramek et al., 2011; Bubici and Papa, 2014). Further, the compound deficiency of JNK1 and JNK2 in mammary epithelial cells promotes tumor formation in KRas/p53 mouse model of breast cancer (Cellurale et al., 2012). Moreover, the inactivation of JNK1 in KO mice spontaneously developed intestinal tumors (Tong et al., 2007; Figure 5D).

These results imply that different JNK isoforms play different roles in tumorigenesis. JNK1, JNK2, and JNK3 may paly opposite role in different cancer cells. Hence, highly specific JNK inhibitors must be employed in clinical use. The regulation of JNK activity during the application of JNK signaling-related medicine in a tissue dependent manner remained to be addressed.

## Conclusion

The JNK signaling pathway is considered as a novel and promising therapeutic target for various biological diseases, understanding its interaction with proteins and their mutual regulation is of great importance. Studies on HEK-293 cell line revealed the interaction between WDR62 and JNKs in the absence of and after stimuli (Wasserman et al., 2010). WDR62 binds to members of JNK family through its C-terminal domain; however, it does not bind to ERK and p38 (Cohen-Katsenelson et al., 2011; Bogoyevitch et al., 2012). MEKK3 forms a complex with WDR62 to synergistically promote the transmission of JNK signal in the nervous system (Xu et al., 2018). Moreover, TAK1 and RAC1 interact with the JNK scaffold protein WDR62 and RAC1 protein POSH (a large number of SH3’s) to form unique JNK protein complexes and participate in brain development especially cortical neuron migration (Zhang et al., 2016). The interactions of WDR62 with JNK pathway components are believed to facilitate participation in various biological functions.

Abnormalities in WDR62 and JNK cause numerous common biological diseases, and the underlying mechanisms are still require intense investigation. WDR62 is required for normal centrosome assembly and centrosomal regulation of the cell cycle (Kodani et al., 2015). JNK regulation is associated with the centrosome throughout the cell cycle, which may require the recruitment of JNK to the centrosome by WDR62 (MacCorkle-Chosnek et al., 2001), and any other hereto unidentified mechanisms. JNK mediates phosphorylation of different substrates to regulate mitosis and brain development. Cell division or cell cycle proteins may act as mediator between WDR62 and JNK. For example, microcephaly proteins regulate CDC25A or CDC25C during cell mitosis (Liu X. et al., 2017). JNK mediates the phosphorylation of CDC25C as well as CDC25A (Gutierrez et al., 2010b). Hence, it is of interest to explore whether CDC25A or CDC25C interacted with and is regulated by WDR62. Several studies suggested that Notch might modulate the JNK signaling pathway (Kim et al., 2005; Cheng et al., 2014). A genetic screen identified WDR62 regulated Notch signaling in the fly wing imaginal disk (Ren et al., 2018). Evidently, further research is needed to understand the regulation between WDR62, JNK and Notch signaling.

The study of WDR62 and JNK signaling in different diseases states related to mitosis and meiosis deregulations pave the way to clinical treatment intended to regulate these processes. Recently, a potential approach to inhibit the proliferation of human breast cancer cells was discovered by using the PLK inhibitor to disrupt the tumor cell centriole (Yeow et al., 2020). WDR62 is a centrosome-associated protein. Its deletion or mutation largely affects the proliferation and differentiation of neural stem and cancer cell, however, whether it is affected by other related signaling pathways in different diseases requires further investigation. In the signaling pathway, JNK’s activation is often affected by external factors, such as cytokines. In brain diseases, JNK activation is often attribute to induction, which leads to hyperphosphorylation of disease-related proteins. Thus, there are numerous drugs that suppress JNK to achieve recovery. In contrast, the development of drugs which can modulate JNK activity shows potential for the treatment of brain diseases as well as cancers and infertility.

The roles of WDR62 and JNK in human diseases dependent on the cellular localization and expression profile of both factors, which are determined by their roles in mitosis and meiosis. Several mouse models were established to study the roles of WDR62 and JNK signaling. However, cell type specific conditional KO mice are lacking, especially regarding different JNK isoforms. Finally, further efforts must be directed toward understanding the different function of WDR62 and JNK signaling in physiological and pathological status. To this end, animal models of human diseases and genetic modification methods will aid in the exploration of underlining mechanisms.

## Author Contributions

DX and YZ conceived the review and wrote, reviewed, edited the manuscript, provided the first draft of the manuscript, and designed the figures. YZ, XZ, JY, LY, HZ, ZX, and DN revised the manuscript. All authors contributed to the article and approved the submitted version.

## Conflict of Interest

The authors declare that the research was conducted in the absence of any commercial or financial relationships that could be construed as a potential conflict of interest.
